# Heterogeneity in Patient-Reported Outcomes following Low-Intensity Mental Health Interventions: A Multilevel Analysis

**DOI:** 10.1371/journal.pone.0099658

**Published:** 2014-09-10

**Authors:** Shehzad Ali, Elizabeth Littlewood, Dean McMillan, Jaime Delgadillo, Alfonso Miranda, Tim Croudace, Simon Gilbody

**Affiliations:** 1 Department of Health Sciences, University of York, York, United Kingdom; 2 Centre for Health Economics, University of York, York, United Kingdom; 3 Primary Care Mental Health Service, Leeds Community Healthcare NHS Trust, Leeds, United Kingdom; 4 Centro de Investigación y Docencia Económicas, Mexico City, Mexico; Federal University of Rio de Janeiro, Brazil

## Abstract

**Background:**

Variability in patient-reported outcomes of psychological treatments has been partly attributed to therapists – a phenomenon commonly known as therapist effects. Meta-analytic reviews reveal wide variation in therapist-attributable variability in psychotherapy outcomes, with most studies reporting therapist effects in the region of 5% to 10% and some finding minimal to no therapist effects. However, all except one study to date have been conducted in high-intensity or mixed intervention groups; therefore, there is scarcity of evidence on therapist effects in brief low-intensity psychological interventions.

**Objective:**

To examine therapist effects in low-intensity interventions for depression and anxiety in a naturalistic setting.

**Data and Analysis:**

Session-by-session data on patient-reported outcome measures were available for a cohort of 1,376 primary care psychotherapy patients treated by 38 therapists. Outcome measures included PHQ-9 (sensitive to depression) and GAD-7 (sensitive to general anxiety disorder) measures. Three-level hierarchical linear modelling was employed to estimate therapist-attributable proportion of variance in clinical outcomes. Therapist effects were evaluated using the intra-cluster correlation coefficient (ICC) and Bayesian empirical predictions of therapist random effects. Three sensitivity analyses were conducted: 1) using both treatment completers and non-completers; 2) a sub-sample of cases with baseline scores above the conventional clinical thresholds for PHQ-9 and GAD-7; and 3) a two-level model (using patient-level pre- and post-treatment scores nested within therapists).

**Results:**

The ICC estimates for all outcome measures were very small, ranging between 0% and 1.3%, although most were statistically significant. The Bayesian empirical predictions showed that therapist random effects were not statistically significantly different from each other. Between patient variability explained most of the variance in outcomes.

**Conclusion:**

Consistent with the only other study to date in low intensity interventions, evidence was found to suggest minimal to no therapist effects in patient-reported outcomes. This draws attention to the more prominent source of variability which is found at the between-patient level.

## Introduction

Variability in outcomes of psychological treatments has been partly attributed to therapists – a phenomenon commonly known as therapist effects (TE). However, despite a wealth of research over more than 20 years, the extent to which TE determine psychotherapy outcomes remains a widely debated topic [1]–[10]. TE may be an important factor to consider when evaluating observed differences between psychotherapy treatments which may be partly explained by differences in the mix of therapists [6], [11]. For example, in a sample of severely depressed patients in the National Institute of Mental Health Treatment of Depression Collaborative Research Program (NIMH TDCRP), Kim et al. (2006) found that observed treatment differences between two psychotherapy interventions disappeared once therapists effects were taken into account [6]. These observations have led some researchers to conclude that ignoring TE in clinical outcome studies may lead to inaccurate effect size estimates and hence result in misleading conclusions [12], [13]. Therapist effects are also be important in understanding the variability in patient-reported outcomes that can partly be explained by heterogeneity of therapists (4, 5).

A prominent source of debate is the wide variation in TE estimates across studies. Some studies report strong evidence of TE (e.g. [4], [6], [9], [14], [15], whilst other studies demonstrate negligible or statistically non-significant TE (e.g. [2], [7], [10], [16], [17]. A meta-analysis of 15 studies involving a heterogeneous mix of patient groups, psychological treatments, therapist numbers and patient sample sizes found that TE accounted for an estimated 8.6% (range 0% to almost 50%) of the variability in patient outcomes following therapy [2]. More recent reviews have reported TE estimates in the range of 5% to 10% [6], [18]. This wide variability across studies raises questions about the relative importance of TE in psychotherapy and the possible explanations for such variability.

Potential explanations for this variability can be broadly divided into three areas: methodological characteristics (study design and analysis), patient and therapist characteristics, and treatment characteristics. Possibly the most contentious issue in this field concerns the methods used to estimate TE. Many authors have argued that the type of statistical modelling applied in these studies can directly influence the estimated therapist effects (e.g. [8], [14], [19], [20]. A good example of this is the re-analysis of the NIMH TDCRP data conducted independently by two research groups, where significant TE of approximately 8% were shown by one group [6], whilst the other group reported little to no evidence of therapist effects [7]. Although both groups analysed data using hierarchical linear modelling (HLM), they differed in how this method was applied. Kim and colleagues (2006) applied a two-level HLM method (patients nested within therapists) which analyses pre-treatment and post-treatment outcome scores only [6], and Elkin and colleagues (2006) used a three-level HLM method (session scores nested within patients, nested within therapist) which analyses longitudinal outcome data to include the effect of change over time [7].

Some authors have argued that the magnitude of TE may vary according to the study design, and can only be reliably estimated from large-scale naturalistic studies [14], [19]. Many TE studies have been conducted within the confines of well-controlled efficacy studies, using a more homogenous patient population and smaller numbers of therapists and patients (e.g. [1], [6], [7], [21]. Such studies have tended to produce mixed results (e.g. [6], [7]). On the other hand, naturalistic studies often include heterogeneous patient populations with larger numbers of both patients and therapists, thus more closely representing routine clinical practice (e.g. [9], [10], [14], [15]). Nevertheless, naturalistic studies have also produced mixed results to date. Although three studies report TE of between 4% and 8% [9], [14], [15], one recent study reported only minimal TE of between 0% and 2% [10]. An additional and potentially important methodological characteristic of these studies concerns the outcome measure used to estimate TE. Some authors have argued that the magnitude of TE estimates may depend on whether the outcome is a generic measure of psychological distress or a disorder-specific measure, and whether outcomes are self-reported or therapist-rated [7], [10]. However, a recent meta-analytic review demonstrated mixed findings for studies that used both the same disorder specific measures (e.g. BDI), more generic measures of distress (e.g. OQ-45) or therapist-rated measures (e.g. GAF) [18].

Besides methodological characteristics, it is possible that the characteristics of the patients and therapists included in a particular study may determine the size and significance of observed therapist effects. Kim and colleagues (2006), for example, found that the magnitude of TE in the NIMH TDCRP was correlated with baseline level of severity [6]. In a meta-analytic investigation, Crits-Christoph and colleagues (1991), found that the use of a treatment manual and more experienced therapists were associated with small TE, whereas more inexperienced therapists and no treatment manual were associated with larger TE [2]. Other studies suggest that the existence of outlier therapists can influence the size of TE, with TE being attenuated or eliminated when outlier therapists are removed (e.g. [6], [21]. Similarly, it is possible that outlier patients may influence the magnitude of TE in a given sample. Saxon and Barkham (2012), for example, argue that very impaired, complex cases inflate TE [22], which may result in smaller therapist effects in samples without this level of case complexity.

Although there is still uncertainty about the extent to which TE are important determinants of therapeutic outcomes, some clear methodological lessons can be drawn from the literature. The magnitude of observed TE is likely to be dependent on a number of factors including the heterogeneity of the patient population (e.g. case-mix), differences in baseline severity of the disorder, the number of therapists involved and patient sample size [20], [22]. Precise TE estimates are more likely to be derived from large naturalistic samples including at least 30 therapists [20]. In addition to controlling for factors that may influence TE, researchers have argued that the use of statistical methods such as multilevel modelling is more appropriate for such data, since they better reflect the natural structure of variability in outcomes (e.g. patients nested within individual therapists) and allow for a partitioning of outcome variance at the patient and therapist levels [22]. In addition, random effects (or mixed) analyses (rather than only fixed effects analyses) better facilitate the computation of standardised therapist variance estimates and the generalisation of findings as relevant to the wider population of therapists [18].

While the current literature provides some insight into the relevance of methodological and patient-therapist characteristics, what has been less studied is the influence of treatment characteristics. Nearly all previous therapist-effect studies have focused on conventional psychotherapeutic interventions, such as cognitive-behaviour therapy (e.g. [4], [6], [7], [10]), interpersonal therapy [6], [7] and psychodynamic therapy (see [2]). Only one previous study has examined therapist effects in brief low-intensity psychological interventions. Almlőv et al. (2011) found little evidence for TE in a study involving low-intensity guided internet-delivered Cognitive Behaviour Therapy [23]. The authors suggest that this is partly explained by similarity of therapists in terms of level of experience and therapeutic orientation. The interpretation of these results, however, is limited by the small patient sample size (119 patients drawn from three individual studies) and small therapist numbers (N = 8).

Brief low-intensity psychological interventions have a number of broad features that differentiate them from more traditional, high-intensity psychotherapeutic interventions [24], [25]. Low-intensity interventions are typically less complex, involve a reduced level of contact with the person receiving treatment, and often use novel forms of delivery (e.g., telephone delivery, computerised Cognitive Behaviour Therapy, provision of self-help material, group treatment) [25]. In addition to the nature of the treatment itself, low-intensity interventions are likely to differ from high-intensity treatments in terms of who provides and who receives the treatment. Low-intensity treatments are typically intended for patients with mild to moderate symptoms and less complex presentations; they are also designed to be delivered by professionals or para-professionals with less extensive training who are likely to have similar level of experience [26]. These characteristics of low-intensity therapy may influence the size and significance of therapist effects. However, there is paucity of evidence on therapist effects in low-intensity psychotherapy. Therefore, the main aim of the current study is to estimate TE in a large naturalistic treatment sample of brief low-intensity treatments for people presenting with common mental health problems such as depression and anxiety.

## Methods

### Ethics statement

Use of anonymous clinical records for this study was approved by an English National Health Service (NHS) research ethics committee, on 15 September 2010, reference 10/H1306/68. Patients provided verbal consent at the earliest contact point with the service which was over the telephone, hence verbal consent was appropriate. Patients were asked whether they agreed to allow their anonymous clinical records to be used for research and audit purposes. Verbal consent was then recorded in the electronic patient clinical record. Anonymous datasets were extracted from electronic clinical records for audit/research purposes, which excluded non-consenting patients. This method was approved by the reviewing NHS ethics committee.

### Study population

This study was based on routinely collected session-by-session patient reported outcome measures (PROMS) for patients accessing treatment for depression and anxiety disorders in a primary care mental health service in Leeds, England. These data are specifically drawn from a cohort of patients who received low intensity (LI) evidence-based interventions recommended by national guidelines [27], [28] and delivered as part of the English Improving Access to Psychological Therapies (IAPT) programme [29]. These interventions included guided self-help based on cognitive behavioural therapy (CBT) principles, one-to-one and group based psycho-education about common mental disorders, and computerised CBT with support by a mental health professional. Such interventions are considered ‘low intensity’ given their brief length (typically under 8 sessions, although a small proportion of patients may have more sessions), and because they are delivered by qualified therapists whose training is briefer and less extensive than that of psychotherapists or clinical psychologists. Therapists in this service completed a standard, 1-year post-graduate qualification specific to the delivery of low intensity treatments and following a national curriculum (e.g. see [26], [30]).

Patients in this cohort were either directly referred to the service by general medical practitioners or encouraged to self-refer. Following referral, all patients attended a 45 minute screening interview with a qualified mental health practitioner. ICD-10 based primary diagnoses were derived from these interviews supplemented by validated case-finding questionnaires. This assessment was used to determine suitability for LI interventions on the basis of a positive screen on depression and/or anxiety measures (described below), and on idiographic data gathered to assess the presenting problem, patient goals and risk factors. Following national guidelines [27], [28], those patients with mild-to-moderate symptoms and functional impairment were deemed suitable for LI interventions and were sequentially allocated to the first available therapist. Although for a minority of patients, their preferences did influence the location of treatment and gender of therapist, there was virtually no selection on the part of therapists. Patients who did not meet criteria for LI interventions (e.g. those with severe, chronic and/or complex conditions) and those who did not improve after LI treatment were ‘stepped up’ to more intensive and lengthier psychotherapy in the service; however, this study only focuses on the sample of patients who received treatment with a therapist qualified to deliver low intensity interventions (including those who were later stepped-up).

### Outcome measures

Data on two commonly used PROMS was available. The Patient Health Questionnaire (PHQ-9) is a nine item self-completed questionnaire commonly used to screen for major depression [31]. This measure is reported to have adequate sensitivity (88%) and specificity (88%) using a cut-off score ≥ 10 [31]. The Generalized Anxiety Disorder scale (GAD-7) is a seven item questionnaire originally developed to detect GAD, although adequate sensitivity (77%) and specificity (82%) estimates have been reported for its capacity to screen for other anxiety disorders including social phobia, post-traumatic stress disorder and panic disorder using a cut-off ≥ 8 [32]. Both measures have been extensively validated and widely used in primary care settings across several countries [33]. Each item in these questionnaires is scored on a 0-3 scale and the scores are summed to give an overall score (range for PHQ-9 = 0-27; GAD-7 = 0-21) with a higher score indicating more severe symptoms.

### Statistical analysis

#### Hierarchical structure

The IAPT data structure is hierarchical and consists of three-levels: level 1 is the ‘visit-level’ for session-by-session PHQ-9 and GAD-7 scores for each patient (with visit 1 being the baseline session), level 2 is the ‘patient-level’ representing scores nested within patients, and level 3 is the ‘therapist-level’ representing patients nested within therapists.

Three-level hierarchical linear mixed (HLM) models were used for primary analysis to evaluate longitudinal variation in patient-reported outcomes based on PHQ-9 and GAD-7 scores. HLM models decompose total variation in health outcomes into variance components attributable to each level, in particular, the therapist-level (level 3). We used unconditional and conditional HLM models for this purpose; the unconditional HLM model is a random intercept model that does not include any covariates (explanatory variables) while the conditional model includes patient-level covariates and decomposes the remaining variation into variance components. For the primarily analysis, data from all patients who had completed therapy was used. A sensitivity analysis included full sample of treatment completers and non-completers (i.e. those who dropped out or were stepped-up to more intense and lengthier psychotherapy). HLM models were separately implemented for PHQ-9 and GAD-7 outcome measures. The analysis was conducted in Stata version 13.1 (Stata Corporation, College Station TX, USA). The statistical models used in the analysis are described in detail below.

#### Unconditional model

Following Raudenbush and Bryk (2002), we present a three-stage formulation of the unconditional HLM model [34].

Visit-level model: The level-1 model for PHQ-9 or GAD-7 scores can be specified as:
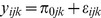
(1)


Here 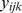 represents the PHQ-9/GAD-7 score for visit *i* for patient *j* who is treated by therapist *k*. The term 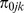 is the patient-level random intercept or the mean PHQ-9/GAD-7 score of patient *j* in therapist *k*. The error term 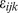 represents the visit-level random deviation of visit *ijk*'s score from the patient-level mean, i.e. the random ‘occasion effect’. 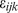 is assumed to be normally distributed with mean 0 and variance σ^2^, i.e. 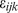 ∼ N(0, σ^2^).

Patient-level model: The patient-level random intercept 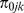 can in turn be modelled as an outcome that varies randomly around the mean score of therapist *k*. This level-2 model can be specified as:
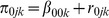
(2)


Here 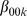 is the mean PHQ-9/GAD-7 score for therapist *k*, and 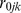 represents the random deviation of patient *jk*'s mean from the therapist mean, i.e. the random ‘patient effect’. As before, 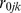 is assumed to be normally distributed with mean 0 and variance 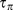.

Therapist-level model: The level-3 model is for the therapist-level intercept 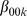 which can be specified as:
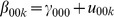
(3)


Here 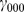 represents the grand mean of PHQ-9/GAD-7 scores in the sample and 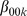 vary randomly around the grand mean which is the only fixed effect in the three-level unconditional model. 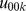 is the therapist-level random effect, i.e. therapist *k*'s random deviation from the grand mean. This random effect is also assumed to be normally distributed with mean 0 and variance 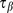.

Combined model: Using the above equations, the combined three-level model can be represented as:
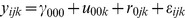
(4)


This is a mixed model as it contains both fixed effect 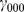 and random effects 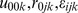.

#### Variance components and intra-cluster correlation coefficient

The three-level model partitions the total variance of the PHQ-9 scores in three components. These are: level-1 variance (σ^2^) attributable to visit-level variability within individuals; level-2 variance (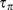) attributable to variability between individuals and within therapists; and level-3 variance (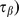 between therapists. The total variance is the sum of these three components:
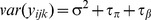
(5)


The proportion of total variance explained by each component can be interpreted as the correlation among observations in each given cluster, also known as the intra-cluster correlation (ICC). In a three level model as the one described above there are three different ICCs:
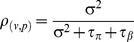
(6)




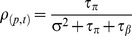
(7)




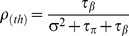
(8)where 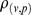 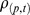 and 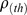 represent the proportion of total variance explained by within patient variability, within therapist variability and between therapists variability respectively. For the current analysis, we are primarily interested in the therapist-level ICC 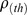.

#### Conditional model

The unconditional model (described earlier) allows estimation of the variance associated with each level. Part of this variance in PHQ-9/GAD-7 scores can be explained by covariates. Hence, we estimate two conditional models:

- Conditional model with quintiles of baseline PHQ-9/GAD-7 score (lowest quintile used as the reference category), age (centred on mean) and gender as covariates.
- The above model plus the following fixed effects variables: visit number for each outcome score (continuous variable); and time since the baseline visit in weeks (continuous variable).

The conditional model can be represented as:
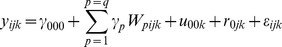
(9)


Here 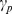 represents the coefficients on covariates *p* nested within patients. The models were estimated using fixed effects at level one and random intercepts for levels 2 (patients) and 3 (therapists). The ICC was calculated to estimate the variance explained at each level.

#### Sensitivity analysis

Three sensitivity analyses were conducted. Firstly, the three-level unconditional HLM analysis for PHQ-9 and GAD-7 was repeated using full sample, including treatment completers and non-completers (i.e. those who dropped out or were stepped-up). Secondly, the three-level unconditional HLM analysis was conducted using only the patients with baseline PHQ-9 score ≥10 (criteria for depression) and baseline GAD-7 score ≥8 (criteria for generalised anxiety disorder) respectively. This was done to evaluate whether the variance attributable to the therapist level is different in the subsample that screened positive for a clinically significant depressive or anxiety disorder (defined based on PHQ-9/GAD-7 thresholds). Finally, following the argument by Wampold and Bolt (2006) [8], we evaluated the robustness of our results by using two-level unconditional and conditional HLM models whereby level-1 was defined as the change score for each patient (i.e. baseline score minus final score for PHQ-9 or GAD-7) and level-2 represented patients nested within therapists. The conditional model included age, gender, baseline severity, total number of visits and total weeks in therapy as covariates. As explained above, the focus is on estimating the variance attributable to the therapist-level.

## Results

### Descriptive statistics

There were 26,177 patients referred to the service during 2008–2010 of which 6,583 patients were allocated to low-intensity psychological interventions based on initial screening. Of these, 2,210 patients had at least one follow-up measure available (either PHQ-9 or GAD-7) after the initial (screening) consultation. Of these, 1,376 patients had completed treatment. There were 38 practitioners who provided low-intensity therapy with mean number of patients per therapist equal to 36.2 (SD = 25.5; range 1–109, with five therapists with <10 patients).

The mean age of patients in the sample was 39.5 (SD = 14.6), the proportion of females was 36.4% and the ethnicity was predominantly white (white: 71.1%; non-white: 5.4%; not known: 23.5%). At patient-level, the mean baseline score (an indicator of symptom severity) in the sample was 11.4 (SD = 5.7) on the PHQ-9 scale and 10.5 (SD = 5.1) on the GAD-7 scale (see Table 1). Based on the commonly used clinical threshold scores of ≥10 for PHQ-9 and ≥8 for GAD-7, 51% patients could be classed as depressed at baseline and 59% as having an anxiety disorder (note: the two conditions often co-existed). Clinical assessment data indicated that the three most commonly recorded primary diagnoses in this sample were depression (37.4%), generalised anxiety disorder (22.7%) and mixed anxiety and depressive disorder (27.9%), with other conditions being less prevalent. At therapist-level, the mean baseline score was 11.4 (SD = 1.1) on the PHQ-9 scale and 10.5 (SD = 0.9) on the GAD-7 scale. The small standard deviation in relation to the mean suggests little variation in baseline severity between therapists [PHQ-9 (p = 0.31) and GAD-7 (p = 0.43)]. This descriptive evidence suggests that allocation of patients to therapists was quasi-random; in other words, there was little evidence of systematic selection of patients at therapist-level.

**Table 1:** Results of unconditional variance components analysis using patient-level PHQ-9 and GAD-7 scores.

Unconditional model	PHQ-9 analysis (patients = 1,359; therapists = 38)				GAD-7 analysis (patients = 1,366; therapists = 38)			
	Estimate	SE	95% CI		Estimate	SE	95% CI	
Fixed effect: intercept	8.13	0.17	7.80	8.46	7.29	0.14	7.02	7.56
Random-effects								
Therapist-level	0.57	0.20	0.29	1.12	0.45	0.18	0.21	0.97
Patient-level	4.24	0.11	4.04	4.45	3.55	0.09	3.37	3.73
Residual	3.72	0.04	3.64	3.80	3.32	0.04	3.25	3.39
Intracluster correlation (ICC%)	Estimate	SE	95% CI		Estimate	SE	95% CI	
Therapist-level	1.0%	0.7%	0.3%	3.8%	0.9%	0.7%	0.2%	3.9%
Patient-level	57.0%	1.4%	54.2%	59.7%	53.7%	1.4%	50.9%	56.5%

**Figure 1:**
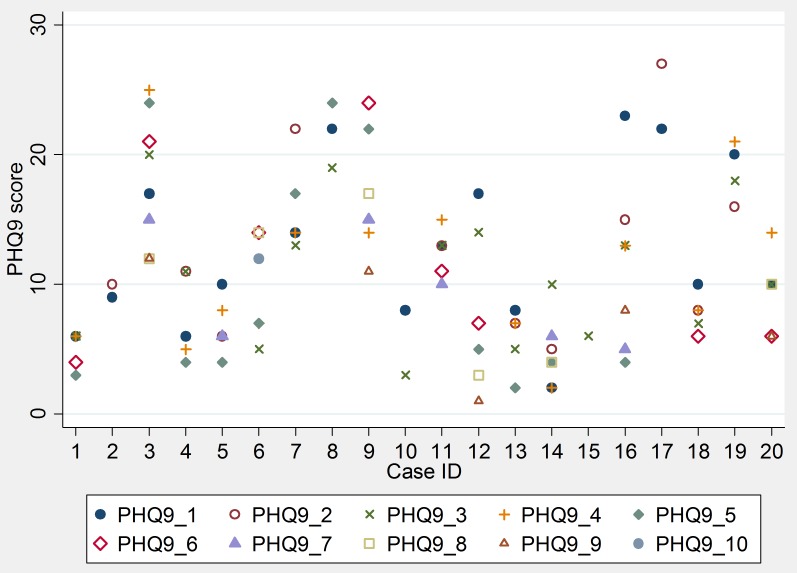
Scatterplot of session-by-session PHQ-9 scores of randomly drawn patients.

Patients attended, on average, 5.2 sessions each (SD = 2.2) and at the end of the therapy had a mean PHQ-9 score of 5.9 (SD = 5.5) and GAD-7 score of 5.4 (SD = 4.7). Figure 1 presents a scatterplot of session-by-session PHQ-9 scores (up to 10 sessions) of a randomly selected sub-sample from the dataset. The figure shows that scores within individuals are highly correlated. A similar clustering pattern was observed in GAD-7 scores (not presented here).

**Figure 2:**
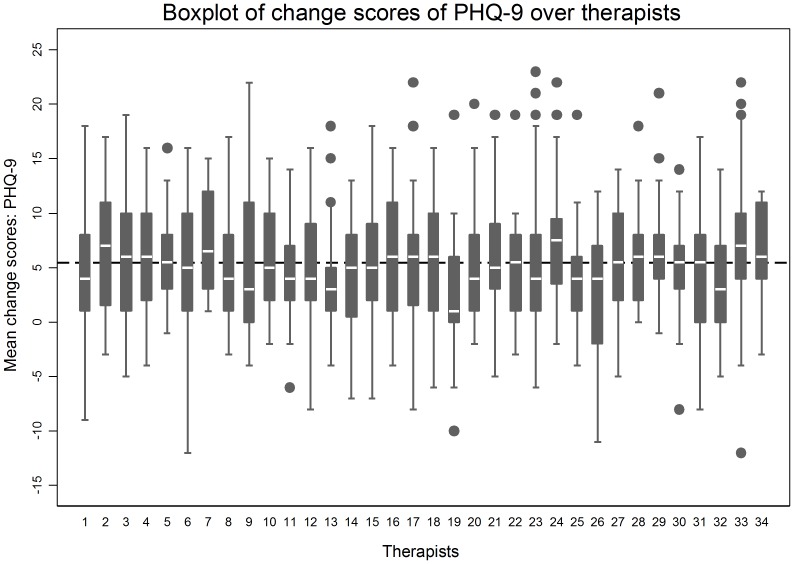
Boxplot of patient-level change scores (difference between baseline and final scores) on the PHQ-9 scale across therapists.

As described above, we also evaluated the patient-level change score (on PHQ-9 and GAD-7 scales separately) over therapists. In the overall sample, the mean patient-level change score on PHQ-9 was 5.5 (SD = 5.5) and on GAD-7 was 5.2 (SD = 5.1). At therapist-level, the mean change score for patients was 5.4 (SD = 1.1) on PHQ-9 and 5.2 (SD = 1.0) on GAD-7. Figure 2 presents a box plot to explore the distribution of change scores in PHQ-9 within and between therapists. The figure shows that, on average, all therapists had improvement in patient-level PHQ-9 scores, although a small number of patients within most therapists had worse scores at the time of discharge. Crucially, the interquartile range of change scores for all therapists overlapped with each other, which denotes little variation between them. A similar distribution of change scores was observed for GAD-7 scores (not presented here).

### Three-level unconditional analysis

The unconditional HLM model decomposed the total variation in patient-level health outcomes (i.e. PHQ-9 and GAD-7 scores) into variation explained by therapists (i.e. between therapist variance 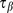), variation explained by patients (i.e. between patient variance,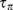) and the residual variance (i.e. within patient varice, σ^2^). Table 1 reports the variance explained at each level and the associated ICC coefficients (i.e. the proportion of variance at each level). The analysis found that of the total variation in patient reported outcomes only around 1% was explained by the therapist level (ICC: PHQ-9 = 1.0%; GAD-7 = 0.9%). The largest share of the total variance was explained at patient level, i.e. between-patient heterogeneity accounted for 57% and 54% of the total variance in PHQ-9 and GAD-7 respectively). This compliments the observation in Figure 1 which showed that scores within individuals were highly correlated. This analysis suggests that, in this cohort of low intensity interventions, only a small proportion of the variation in outcomes can be attributed to therapists. In other words the therapist effect is very small, which would indicate comparable and fairly uniform average outcomes between different therapists.

**Figure 3:**
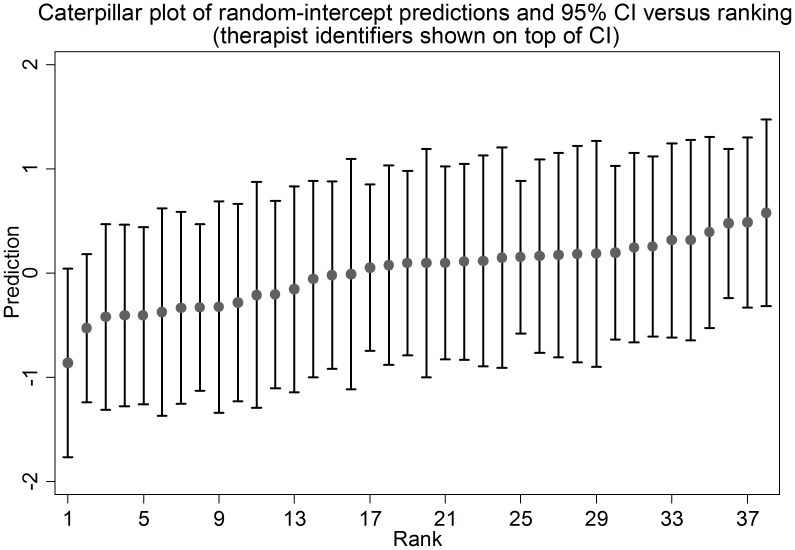
Caterpillar plot of predicted therapist-level random intercept and 95% CI versus average performance benchmark.

Bayesian empirical predictions were used to predict values of random intercepts for each therapist. The random intercepts represent the relative effectiveness of each therapist in improving health outcomes (either PHQ-9 or GAD-7) compared to the average therapist in the sample. The therapist random intercepts are ranked and presented with their 95% CI in Figure 3 (caterpillar plot). Therapists with better than average outcomes have negative intercepts and hence are in the bottom left corner of the plot and the lowest performing therapists in the sample have positive intercepts and are in the top left. The 95% CI of all random intercepts cross zero which suggests that therapists are not statistically significantly different from the average therapist in the group. Moreover, all therapist-level random intercepts overlap which corroborates the finding that there was no evidence of statistically significant differences in performance between therapists.

### Three-level conditional analysis

The conditional multilevel models control for imbalance in patient-level covariates and attribute the remaining variance to the three levels. The first conditional model included fixed effects variables representing quintiles of baseline PHQ-9/GAD-7 scores, age and gender. As expected, patients in quintiles with higher baseline scores (or more severe symptom severity) were found to have higher scores at successive visits (compared to patients in lower baseline quintiles). Patient's age and gender were not found to have a statistically significant relationship with health outcomes. More importantly, the ICC for therapist-level was 0.4% and 0.6% in PHQ-9 and GAD-7 analyses respectively (Table 2), and most of the remaining variance was attributable to between-patient heterogeneity and within-patient random error. Notice, however, that the therapist random effect is statistically non-significant at 5% in the PHQ-9 model but significant in the GAD-7 model.

**Table 2:** Results of conditional multilevel analysis using patient-level PHQ-9 and GAD-7 scores.

Conditional model 1	PHQ-9 analysis (patients = 1,174; therapists = 37)				GAD-7 analysis (patients = 1,190; therapists = 37)			
	Estimate	SE	95% CI		Estimate	SE	95% CI	
Fixed effects								
Baseline PHQ-9/GAD-7 quintiles								
Baseline quintile 2	1.94	0.33	1.29	2.59	2.00	0.28	1.45	2.54
Baseline quintile 3	3.78	0.31	3.18	4.37	3.02	0.28	2.47	3.56
Baseline quintile 4	6.32	0.32	5.69	6.95	4.51	0.29	3.94	5.08
Baseline quintile 5	9.69	0.33	9.03	10.34	7.29	0.27	6.76	7.83
Age of the patient	0.00	0.01	−0.01	0.02	0.00	0.01	−0.01	0.02
Gender (Female = 1)	0.11	0.21	−0.31	0.53	0.03	0.19	−0.34	0.40
Intercept	3.90	0.27	3.38	4.43	4.07	0.23	3.63	4.51
Random effects								
Therapist-level	0.29	0.18	0.08	0.97	0.33	0.15	0.14	0.78
Patient-level	2.86	0.09	2.69	3.04	2.53	0.08	2.38	2.69
Residual	3.61	0.04	3.53	3.70	3.28	0.04	3.20	3.36
Intracluster correlation (ICC%)	Estimate	SE	95% CI		Estimate	SE	95% CI	
Therapist-level	0.4%	0.5%	0.0%	4.3%	0.6%	0.6%	0.1%	3.5%
Patient-level	38.7%	1.6%	35.5%	41.9%	37.7%	1.6%	34.5%	40.9%

Conditional model 2	PHQ-9 analysis (patients = 1,174; therapists = 37)				GAD-7 analysis (patients = 1,127; therapists = 37)			
	Estimate	SE	95% CI		Estimate	SE	95% CI	
Fixed effects								
Baseline PHQ-9/GAD-7 quintiles								
Baseline quintile 2	2.05	0.35	1.36	2.75	2.16	0.30	1.56	2.75
Baseline quintile 3	4.03	0.33	3.38	4.68	3.20	0.30	2.62	3.79
Baseline quintile 4	6.68	0.35	6.00	7.36	4.83	0.31	4.21	5.44
Baseline quintile 5	10.26	0.36	9.54	10.97	7.67	0.30	7.09	8.26
Age of the patient	0.00	0.01	−0.02	0.01	0.00	0.01	−0.01	0.01
Gender (Female = 1)	0.26	0.23	−0.19	0.72	0.18	0.21	−0.23	0.58
Visits (continuous)	-0.66	0.06	−0.78	−0.55	−0.54	0.05	−0.65	−0.44
Time since first visit (in weeks)	0.04	0.02	−0.01	0.09	0.01	0.02	−0.03	0.05
Intercept	5.64	0.31	5.04	6.24	5.60	0.26	5.10	6.11
Random effects								
Therapist-level	0.36	0.19	0.13	0.99	0.35	0.16	0.15	0.85
Patient-level	3.10	0.09	2.92	3.29	2.74	0.09	2.57	2.91
Residual	3.19	0.04	3.10	3.27	2.95	0.04	2.87	3.03
Intracluster correlation (ICC%)	Estimate	SE	95% CI		Estimate	SE	95% CI	
Therapist-level	0.7%	0.7%	0.1%	4.8%	0.8%	0.7%	0.1%	4.3%
Patient-level	49.0%	1.8%	45.6%	52.5%	46.6%	1.8%	43.1%	50.2%

In the second conditional model, we added (in addition to the covariates in the above model) fixed effects dummy variables for each visit, except the first therapy visit (reference category) to explicitly control for changes in outcomes over the course of treatment. The results were similar to earlier models and the ICC was found to be <1% for therapist level in both models (Table 2).

### Sensitivity analyses

To evaluate the robustness of our findings, three types of sensitivity analyses were conducted [Tables 3 and 4]. The first analysis included full sample of patients (including non-completers of treatment) and the second analysis included only those patients with PHQ-9 scores ≥10 or GAD-7 score ≥8 (i.e. patients with baseline scores above the thresholds for depression or anxiety disorders). These analyses found that the variance explained at the therapist level was only between 0.2% and 1.2%. A further sensitivity analysis explored variance attributable to therapists in two-level unconditional and unconditional analyses (i.e. patient-level change score nested within therapists) and found therapist-level ICC to be between 0% and 1.3% for the PROMs.

**Table 3:** Sensitivity analysis I: multilevel analysis using [1] full sample and [2] patient with PHQ-9≥ 10 and GAD-7≥8.

[1] Full sample analysis (both treatment completers and non-completers)	PHQ-9 analysis (patients = 2,190; therapists = 38)				GAD-7 analysis (patients = 2,197; therapists = 38)			
	Estimate	SE	95% CI		Estimate	SE	95% CI	
Fixed effect: intercept	9.33	0.14	9.05	9.61	8.32	0.11	8.11	8.53
Random-effects								
Therapist-level	0.45	0.17	0.21	0.95	0.22	0.20	0.04	1.242
Patient-level	4.82	0.09	4.64	5.01	4.04	0.08	3.89	4.204
Residual	3.67	0.04	3.60	3.74	3.32	0.03	3.25	3.384
Intracluster correlation (ICC%)								
Therapist-level	0.5%	0.4%	0.1%	2.4%	0.2%	0.3%	0.0%	5.4%
Patient-level	63.5%	1.1%	61.4%	65.6%	59.8%	1.1%	57.6%	62.0%

[2] Patients with PHQ-9≥10/GAD-7≥8	PHQ-9≥ 10 (patients = 703; therapists = 37)				GAD-7≥8 (patients = 811; therapists = 37)			
	Estimate	SE	95% CI		Estimate	SE	95% CI	
Fixed effect: intercept	10.29	0.21	9.87	10.71	8.54	0.17	8.21	8.88
Random-effects								
Therapist-level	0.63	0.27	0.28	1.45	0.53	0.22	0.23	1.198
Patient-level	4.06	0.15	3.78	4.36	3.37	0.12	3.15	3.613
Residual	4.10	0.06	3.98	4.22	3.57	0.05	3.47	3.670
Intracluster correlation (ICC%)								
Therapist-level	1.2%	1.0%	0.2%	6.0%	1.1%	0.9%	0.2%	5.7%
Patient-level	50.2%	2.0%	46.2%	54.1%	47.8%	1.9%	44.0%	51.6%

**Table 4:** Sensitivity analysis: two-level analysis of change scores using [3] unconditional model and [4] conditional model.

[3] Unconditional two-level analysis using change scores	PHQ-9 analysis (patients = 1,194; therapists = 37)				GAD-7 analysis (patients = 1,206; therapists = 37)			
	Estimate	SE	95% CI		Estimate	SE	95% CI	
Fixed effect: intercept	5.44	0.18	5.08	5.80	5.17	0.15	4.88	5.461
Random-effects								
Therapist-level	0.51	0.26	0.19	1.38	2.16E-07	8.79E-07	7.51E-11	0.001
Residual	5.44	0.11	5.22	5.66	5.12	0.10	4.92	5.33
Intracluster correlation (ICC%)								
Therapist-level	0.9%	0.9%	0.1%	6.1%	1.8E-15	0.0E+00	1.8E-15	1.8E-15

[4] Conditional two-level analysis using change scores	PHQ-9 analysis (patients = 1,184; therapists = 37)				GAD-7 analysis (patients = 1,196; therapists = 37)			
	Estimate	SE	95% CI		Estimate	SE	95% CI	
Fixed effects								
Baseline PHQ-9/GAD-7 quintiles								
Baseline quintile 2	2.67	0.42	1.85	3.49	1.99	0.36	1.28	2.70
Baseline quintile 3	4.54	0.39	3.77	5.31	4.28	0.36	3.57	4.99
Baseline quintile 4	6.49	0.41	5.68	7.30	6.46	0.39	5.69	7.22
Baseline quintile 5	8.21	0.43	7.38	9.05	7.68	0.36	6.97	8.38
Age of the patient	0.00	0.01	−0.02	0.02	0.00	0.01	−0.02	0.01
Gender (Female = 1)	−0.23	0.28	−0.77	0.31	0.17	0.25	−0.32	0.66
Total number of visits	0.31	0.08	0.16	0.46	0.27	0.07	0.13	0.41
Total weeks in therapy	−0.02	0.02	−0.06	0.02	−0.02	0.02	−0.05	0.02
Intercept	0.11	0.59	−1.05	1.27	0.29	0.53	−0.75	1.33
Random effects								
Therapist-level	0.51	0.21	0.22	1.15	0.47	0.20	0.20	1.10
Residual	4.55	0.09	4.37	4.74	4.13	0.09	3.97	4.30
Intracluster correlation (ICC%)								
Therapist-level	1.2%	1.0%	0.2%	6.0%	1.3%	1.1%	0.2%	6.7%

## Discussion

This is the first large study in a naturalistic low-intensity psychotherapy setting that evaluated the contribution of therapists in variability of patient-reported outcome measures. The study had a sample size of 1,376 primary care patients treated by 38 therapists (an average of 36 patients per therapist). The study used three-level hierarchical linear model to estimate therapist effects, but also evaluated two-level model in a sensitivity analysis. The analysis was conducted using the overall sample, and, to assess the robustness of results, using a sub-sample with initial severity above the threshold scores for depression or anxiety based on PHQ-9 or GAD-7 scores, respectively.

This analysis of therapist effects in routine low intensity psychological interventions found TE estimates in the region of 0% to 1.3%. While statistically significant, these estimates were substantially smaller than those typically reported in more traditional high-intensity psychotherapeutic interventions (e.g.,5% average TE estimate reported by Baldwin & Imel, 2013 [18]. The results are, however, consistent with the only previous study of therapist effects in low-intensity treatments [23]. Our results suggest that TE are less prominent in brief low intensity interventions.

However, as outlined in the introduction, TE estimates can be influenced by a range of methodological and sample characteristics. It is therefore important to first examine if study design and data analysis methods may account for the small observed therapist effects in this study. While the main analysis used a three-level hierarchical model (consistent with previously published studies), the analysis was repeated using a two-level model (using only pre- and post-treatment scores) in a sensitivity analysis; this analysis also resulted in small (<1%) though statistically significant TE estimates. Therefore the results seem robust to analytical methods and are unlikely to be an artefact of the type of HLM used.

The current study used data from a large, naturalistic cohort. A number of authors have recommended this design as the most appropriate one to identify therapist effects (e.g.[10], [14], [19] and large effects have been found in a number of such designs (e.g. [9], [10], [14], [15]. The review by Baldwin and Imel (2013), in fact, found that naturalistic cohorts reported significantly larger therapist effects compared to tightly controlled randomised controlled trials (7% vs. 3%) [18]. This aspect of the design, therefore, is also unlikely to account for the small TE estimates.

This study used depression and anxiety outcome measures which broadly matched the clinical diagnosis of patients in the sample. It could be speculated that these measures may not be sensitive enough to capture important variations in effect across caseloads. However, the existing literature reveals large TE estimates using much more generic measures of effect such as the Global Assessment of Functioning, Global Distress Scale, etc [18]. Furthermore, the measures applied in this study have been shown to be sensitive enough to reveal considerable variations in outcome when comparing outcome estimates clustered by specific psychological services (e.g. see [35]). It therefore seems unlikely that the choice of outcome measures entirely provides an alternative explanation for the small therapist effects found in this study.

Although the present results are unlikely to be artefacts of data analysis methods, alternative explanations for the modest TE may include the influence of patient, therapist and treatment characteristics in a low-intensity psychotherapy setting. Consistent with Kim et al. (2006) [6], our sensitivity analyses provide some evidence that baseline severity is modestly associated with the magnitude of TE, since these estimates increased marginally once we excluded cases with sub-clinical baseline scores. Saxon and Barkham (2012) offer a potential clinical interpretation for this association [22]. In an analysis of a large naturalistic cohort of more than 10,000 patients treated by over 100 therapists, they found that greater severity of symptoms was associated with increased TE, such that higher severity and risk was associated with poorer outcomes. Since low intensity treatments are usually offered to patients with mild-to-moderate mental health problems with relatively low risk factors this may naturally attenuate the extent of TE. Besides initial case severity, other characteristics of case complexity (e.g., level of co-morbidity, axis II difficulties) or changes in medication use may influence TE estimates. However, because IAPT routine data does not include measures of case complexity or medication use, we could not explore the influence of these characteristics. Therefore, it is possible that both the low initial severity and case complexity of the low-intensity sample have contributed to the small TE observed in this study.

As described earlier, there was substantial heterogeneity in terms of the diagnoses and presenting problems of clients in this sample. As others have argued [18], it is likely that large within-therapist variability resulting from clinical heterogeneity may overshadow between-therapist variability. In other words, outcome differences between therapists are found to be less significant compared to the wide variation in outcomes within therapists. In this study, the within-therapist (patient-level) variability accounted for most of variance in final treatment outcome (54–57% in the unconditional model, 37–39% in the conditional model).

Another plausible explanation for the modest TE in this sample relates to the level of standardisation of clinical practice that is typical of this cohort of low intensity therapists. Low intensity therapists in the English IAPT programme are trained to offer brief, structured and highly standardised interventions based on common bibliographic materials, and typically work under considerable levels of case-management scrutiny [30], [36]. This, in turn, reduces heterogeneity of clinical practice. High levels of standardisation of treatment such as adherence to treatment manuals have been shown to attenuate TE [2], [10], and TE estimates from tightly controlled efficacy studies also tend to be smaller [18]. The only other study examining TE in low intensity treatments [23] also applied highly standardised interventions relying on computerized CBT. It remains to be seen whether the accumulation of future studies examining TE in low intensity therapy confirm the standardisation of treatment as an important determinant of between-therapist variance.

### Clinical implications

This study concurs with prior evidence on low-intensity psychotherapy that a small proportion of variation in patient outcomes can be attributed to the therapist level, and that between-therapist variability is very modest by comparison to that observed in conventional high-intensity psychotherapies. The findings of this study suggest that when therapists have similar level of training, work under regular supervision and follow pre-specified treatment protocols to deliver low-intensity interventions, there is likely to be less variability in outcomes between therapists. However, it is quite possible that TE estimates could be larger in other services due to variability in experience, supervision and monitoring procedures. Moreover, the findings of low-intensity psychotherapy for patients with less severe common mental health conditions may not apply to outcomes in severe mental health conditions where there tends to be greater variability in baseline severity. Therefore, the well-established practice of systematic collection and monitoring of patient outcomes at therapist-level as means of quality control is still likely to be important, especially in less standardised services. Lessons from the wider literature in this field suggest that quality control strategies should pay particular attention to cases with high baseline severity and risk, since these cases seem more liable to poor outcomes and may require matching to highly skilled ‘outlier’ therapists [22]. Providing feedback about such ‘risk cases’ to the relevant therapists may in itself be a useful method to improve the quality of treatment and outcomes for some patients [37], [38].

It is clear that not every patient benefits from treatment (this applies equally to low and high intensity treatments); however, this study has shown that patient-level variability is the key factor that explains variation in outcomes. This implies that low-intensity therapists should be interested in understanding patient heterogeneity to reduce variability in outcomes. Future studies could, for example, investigate which patient characteristics (besides the ones investigated here) can explain within-therapist variability to allow more patient-centred therapeutic approaches to emerge.
